# Assessing Walking Strategies Using Insole Pressure Sensors for Stroke Survivors

**DOI:** 10.3390/s16101631

**Published:** 2016-10-01

**Authors:** Mario Munoz-Organero, Jack Parker, Lauren Powell, Susan Mawson

**Affiliations:** 1 Telematics Engineering Department, Universidad Carlos III de Madrid; Av. Universidad, 30, 28911 Leganes, Spain; 2 School of Health and Related Research, University of Sheffield; Regent Court, 30, S1 4DA Sheffield, UK; jack.parker@sheffield.ac.uk (J.P.); l.a.powell@sheffield.ac.uk (L.P.); s.mawson@sheffield.ac.uk (S.M.)

**Keywords:** insole pressure sensors, stroke survivals, machine learning, rehabilitation, walking strategies

## Abstract

Insole pressure sensors capture the different forces exercised over the different parts of the sole when performing tasks standing up such as walking. Using data analysis and machine learning techniques, common patterns and strategies from different users to achieve different tasks can be automatically extracted. In this paper, we present the results obtained for the automatic detection of different strategies used by stroke survivors when walking as integrated into an Information Communication Technology (ICT) enhanced Personalised Self-Management Rehabilitation System (PSMrS) for stroke rehabilitation. Fourteen stroke survivors and 10 healthy controls have participated in the experiment by walking six times a distance from chair to chair of approximately 10 m long. The Rivermead Mobility Index was used to assess the functional ability of each individual in the stroke survivor group. Several walking strategies are studied based on data gathered from insole pressure sensors and patterns found in stroke survivor patients are compared with average patterns found in healthy control users. A mechanism to automatically estimate a mobility index based on the similarity of the pressure patterns to a stereotyped stride is also used. Both data gathered from stroke survivors and healthy controls are used to evaluate the proposed mechanisms. The output of trained algorithms is applied to the PSMrS system to provide feedback on gait quality enabling stroke survivors to self-manage their rehabilitation.

## 1. Introduction

The use of shoe insole pressure sensors for the analysis of gait is increasing, providing both researcher and clinician better efficiency, flexibility and cost reduction [1]. Using insole pressure sensors, different patterns and strategies for executing different functional tasks can be assessed and a comparison between different time points and normal data could be the basis for their use in areas such as rehabilitation, pre-habilitation or sport training [2].

Insole pressure sensors have already been used in a number of different areas. The authors in [3] used them for learning Tai-Chi Chuan. Their use in ulcer prevention is presented in [4] where a low cost and flexible plantar pressure monitoring system is presented for everyday use to prevent pressure ulcers. Furthermore, pressure sensors are used in [5] for monitoring elderly people who have a high risk of falling and other mobility problems. Among the different applications, “smart” or “intelligent” insoles may be used to assess long-term chronic conditions that affect the elderly population such as Dementia, Parkinson’s disease, Cancer, Cardiac Disease, Diabetes and Stroke [6]. 

The global incidence of stroke is set to escalate from 15.3 million to 23 million by 2030 and it is one of the largest causes of disability worldwide [7,8]. Approximately two out of three individuals post-stroke experience impaired walking ability with subsequent falls risk and associated social isolation [9,10]. Therefore, the relearning of walking is a major component of stroke rehabilitation with a self-managed rehabilitation paradigm being advocated by many [11]. Assessing walking strategies from the data captured from insole pressure sensors, and using automatically computed distortion indexes, could be a valuable tool to help stroke survivors to improve their gait. Furthermore, feedback from the insole can be used to motivate self-managed rehabilitation post discharge from the health provider [12].

Different types of insoles with different numbers of sensors have been used in previous studies to detect gait related features from stroke survivors. The authors in [13] present a tailor made 3D insole for plantar pressure measurement, comparing it with conventional flat insoles. The authors in [14] use a 32-sensor insole able to replicate the shape of the ground reaction force and ankle moment in a stroke patient who has regained a more normal gait. The results present some limitations for stroke patients with impaired gait. The authors state that subsets of sensors can be evaluated in order to ultimately identify an optimum set of sensors for determining kinetic variables necessary to classify presence or absence of a particular gait abnormality or other pathology.

The pressure patterns over time have been used in several studies to characterize the gait for post-stroke patients. The authors in [15] evaluated changes in the centre of pressure displacement in individuals with stroke with and without a foot drop stimulator. Differences are reflected both in the location and the amplitude of the patterns. The authors in [16] investigated the association between the weight-bearing ratio (WBR) and gait ability of a paretic lower limb while walking using a shoe-type load-measuring apparatus. Another study capturing pressure variations during walking can be found in [17].

In order to provide motivation for self-managed rehabilitation of gait for post-stroke patients, it is important to give user-adapted feedback to each individual, integrating the key sources of self- efficacy [12,18]. The work in [19] presents a device that measures the weight pressure distribution that the a patient exerts on each foot, in addition to the gait time, swing time, and stance time of each leg while walking. Based on real time information, biofeedback is given by means of auditory, and unpleasant electro-tactile stimulation to actively correct gait asymmetry. A similar feedback mechanism providing auditory or tactile feedback to the individual wearing the platform is presented in [20]. In these studies, feedback is based on the amount of time spent in stance phase on each foot, as measured by the pressure sensors embedded into the insoles. The number of steps required to assess different gait related parameter is presented in [21], where the authors determined the test-retest reliability of insole assessment for curved as well as linear trajectories, and estimated the minimum number of steps required to obtain excellent reliability for each output variable.

In 2007, the SMART consortium [22] commenced a programme of research to develop and evaluate an Information Communication Technology (ICT) enhanced Personalised Self-Management Rehabilitation System (PSMrS) [12,23,24,25] for stroke rehabilitation. The intervention model for the stroke system was based around a rehabilitation paradigm underpinned by theories of motor relearning and neuroplastic adaptation, motivational feedback, self-efficacy and knowledge transfer [26,27,28,29].

**Figure 1:**
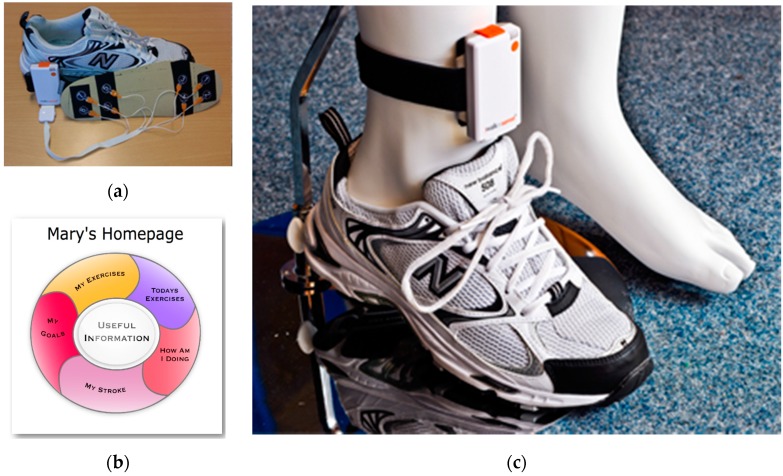
The prototype PSMrS for stroke survivors. The Walk-in-sense device (Kinematix, Porto, Portugal) (**a**); the user interface (**b**) and attachment of devices to lower limb on a manikin (**c**).

The PSMrS is a prototype rehabilitation system with wearable sensor technology within an “intelligent shoe”, developed to provide feedback on gait quality, specifically symmetry, thereby enabling stroking survivors to self-manage their rehabilitation [12] (see Figure 1). The major contribution of this paper is the assessment and validation of the automatic detection and characterization of walking strategies based on the use of the “intelligent shoe” in Figure 1 and its integration in the PSMsS system for an Information Communication Technology (ICT) enhanced Personalised Self-Management Rehabilitation. The scope of the paper is to assess and evaluate how accurately and effectively walking strategies can be detected and monitored by the system for self-rehabilitation and how well in terms of correlation and coefficient of determination a commonly used index (the Rivermead Mobility Index, RMI) can be assessed based on auto-detected features.

The effectiveness of interventions based on an Information Communication Technology (ICT) enhanced Personalised Self-Management Rehabilitation System (PSMrS) depends on the way the user is motivated and the type of feedback provided whether this be intrinsic feedback or extrinsic feedback [18,30,31]. The PSMrS uses a personal area network that comprises of the intelligent insole that transmits data in real time via a Bluetooth channel connected to a mobile phone. The insole comprises a network of eight force sensitive resistors per foot/insole and samples data at a frequency of 100 Hz at a resolution of 8 bits. The data are captured in real time and uploaded to a server for further analysis for each walking activity with the intention of feeding this back to the stroke survivor as summative data.

Sources of self-efficacy, a key pre-requisite for self-management, are obtained directly from the information and feedback a user receives from the performance of a task [32]. Four key sources of self-efficacy have been identified within the literature [33]:

- Mastery experiences: Gaining confidence in achieving and accomplishing tasks.
- Modelling: The observation of similar individuals achieving and accomplishing tasks through direct observation or through written and visual material.
- Interpreting physiological signs: Having the ability and confidence to interpret symptoms and changes in symptoms such as, mobility, weakness, stiffness, and fatigue.
- Feedback and persuasion: The provision of some recognition of their performance and progress from personal achievement as well as significant others, i.e., family members.

In this study, we used the insole pressure sensors from the PSMrS to get a deeper insight about the different strategies used by stroke survivors based on the assessment of gait parameters, on pressure graphs and forefoot to heel parameter extraction. The overarching aim was to explore the strategies in order to inform further developments of the motivational feedback screens within the PSMrS thereby proving the four sources of self-efficacy described above. As an initial pilot, we studied 14 stroke survivors and 10 healthy controls who performed a 10-m walk test (repeated six times) with resting pauses to record the pressure signals over time in both insoles. The controls were used to provide objective parameters that could be integrated into the PSMrS to provide a reference cluster that can be used as a source to compare self-efficacy for the users.

## 2. Method

### 2.1. Aim

The aim of the study presented in this paper was to determine how automatically computed features could be used to assess walking strategies using the intelligent insole with stroke survivors and healthy controls aged 45 years and over in order to provide feedback on these strategies to enhance self-efficacy, self-management and thereby promote motor relearning. Normal patterns from the healthy controls being used in this novel system to provide comparisons data for the system. The scope of the paper is to assess and evaluate how accurately and effectively walking strategies can be detected and monitored by the system for self-rehabilitation and how well in terms of correlation and coefficient of determination a commonly used index (the Rivermead Mobility Index, RMI) can be assessed based on auto-detected features. 

We undertook an observational, cohort pilot study of 14 stroke survivors and 10 healthy controls who both performed a 10-m walk test (repeated 6 times) with resting pauses to record the pressure signals over time in both insoles.

### 2.2. Inclusion Criteria

The inclusion criteria took into account the degree of rehabilitation that each candidate participant had already taken in order to select patients in different rehabilitation stages.

The inclusion criteria for the stroke survivors were:

- A definite diagnosis of stroke (self-reported);
- Able to give informed consent;
- Able to walk (with or without walking aids); and
- Individuals with self-reported walking difficulties as a result of their stroke.

The exclusion criteria used for the stroke survivors were:

- Unable to speak or comprehend written English;
- Unable to give informed consent; and
- Medically unstable, or other neurological, neuromuscular, or orthopaedic disorders that would interfere with task performance (self-reported).

The inclusion criteria for the healthy controls were:

- 45 years of age or over;
- No conditions effecting their walking, e.g., back pain, hip conditions, and arthritis in the knees;
- Able to give informed consent;
- Able to speak and comprehend written English;
- No history of stroke; and
- Medically stable, and no other neurological, neuromuscular, or orthopaedic disorders that would interfere with task performance (self-reported).

### 2.3. Recruitment

Members of the research team visited a number of Stroke Community Teams in Sheffield and provided information sheets to the group members. Interested group members then contacted the research team to confirm their eligibility and arrange an appointment to take part in the study. To recruit the healthy controls, an email was sent to University staff with the information sheet attached and the inclusion criteria. Individuals were screened for eligibility via email correspondence. Volunteers were University staff and eligible friends/family members of university staff. 

### 2.4. Setting

The study took place in the Centre for Assistive Technology and Connected Healthcare (CATCH) HomeLab, in the University of Sheffield. The CATCH HomeLab mimics the home environment, allowing participants to experience a setting that represents their day-to-day lives at home. The HomeLab provided a clear, flat, levelled surface for participants to carry out their walks.

### 2.5. Procedures

Stroke survivors and healthy subjects walked a distance of six meters wearing two intelligent insoles, one in each shoe. Different sizes for the insoles were available in order to accommodate the insole to the participants’ shoes in an accurate way. Participants were asked to wear outdoor shoes for their visit (to accommodate the insoles). Data collection for each participant began from a seated position. They then stood up, executed the walk, and sat back down after walking around 10 m.

Participants were video recorded from the waist down to allow us to visually link the physical movement of participants to the data generated by the Intelligent Shoe. These two pieces of information (intelligent insole data and video recordings) were then synchronised. In addition, participants in the stroke group were asked to complete the Rivermead Mobility Index (RMI), which has been used and validated for use with a post stroke population. It involved the participants answering yes or no to 14 questions on a questionnaire and to complete one observation (standing up for 10 s). The RMI was administered by a physiotherapist [34].

Each insole is equipped with 8 pressure sensors (Figure 1). The sensors were sampled at 100 Hz (one sample each 10 ms). The insoles were powered by a LiPo (Lithium polymer battery) with 10 h of continuous usage battery life which was pre-charged before the start of the measurements. Each participant was asked to stand up and sit down before starting the recording of the pressure patterns to validate the proper functioning of all sensors. The sensors transmitted the measurements in real time to a central computer using Bluetooth Low Energy. The central computer ran a software tool from Kinematix [35] to store the raw data. The raw data were exported to a comma separated values (CSV) file for post-processing and feature detection. A pre-filtering of the data was performed in order to isolate each step between inactivity segments. The authors cross-validated the automatically pre-detected steps and manually corrected miss-classified elements to ensure that feature computing was only based on the stance phase of the recorded steps. The following features have been computed based on the post-processed data from the sensors:

- Time in which the average pressure on the heel is bigger than the 80% of the maximum heel pressure;
- Maximum forefoot pressure;
- Ratio between the forefoot pressure at the point of maximum heel pressure and the maximum forefoot pressure;
- Anterior–posterior pressure pattern length;
- Ratio between the maximum forefoot pressure and the maximum heel pressure;
- Anterior–posterior pressure length and location; and
- Lateral pressure length and location.

Optimal results for classification have been obtained with the J48 algorithm. The J48 algorithm is an open source implementation in Java of the C4.5 algorithm in the Weka data mining tool [36,37]. The algorithm generates a decision tree which selects the feature that maximizes the information gain at each node of the tree. The information gain is measured as the difference in the entropy [38] between the posterior and prior distributions conditioned to each feature. 

### 2.6. Ethical Considerations

Ethics approval was provided by the ethics committee at the University of Sheffield. The intelligent insoles are thin and would not affect participants’ gait. However, during their walks, they were supervised throughout. Participants also used any walking aids that they use routinely, e.g., a walking stick. All participants were aware that there were a minimum number of other people in the room present when they were asked to walk. 

### 2.7. Consent

All participants received an information sheet a minimum of 1 week before data collection. They were given the opportunity to ask questions before data collection begun. Participants gave informed consent by signing a consent form on the day data was collected. Participants had the option to consent to be contacted in the future for future related research. They did not have to agree to this to take part in this study. 

### 2.8. Stroke Survivor Participants Demographics

Data were recorded for 14 stroke survivors; see Table 1 for subject’s demographics. Participants included 7 males and 7 females, with a mean age of 66.43 and standard deviation of 11.51. 

### 2.9. Control Participants Demographics

Data were recorded for 10 healthy controls; see Table 2 for subject’s demographics. Participants included 3 males and 7 females, with a mean age of 48.8 and standard deviation of 4.08.

## 3. Walking Strategies and Selected Parameters

Walking ability is affected after stroke (two out of three individuals post-stroke experience impaired walking ability [9,10]). From a clinical perspective there are a number of characteristics and walking strategies that are commonly utilized by stroke survivors, if these strategies continue without intervention, musculoskeletal changes such as muscle shortening, muscle imbalance and muscle weakness would be the short term consequence with a significant impact on functional activities in the long term. In order to achieve our aims, we needed to assess how well automatic detected features could be integrated within the PSMrS for self-rehabilitation management the overarching aim being to prevent such short and long term consequences developing after a stroke. In this section, some features are defined. The use of the pressure sensors for validation is left to the next section.

### 3.1. Heel Walking Strategy

A lack of dorsiflexion in the hemiplegic leg causes long high pressure times on the heel. In order to assess this walking strategy, we considered the following parameters:

- Time in which the average pressure on the heel is bigger than the 80% of the maximum heel pressure; and
- Maximum forefoot pressure.

Parameter A measures the length of the heel contact (when the heel is most active). Parameter B was used to compute the ratio B/A, which can be used to detect this walking strategy in order to assess abnormal low maximum values of maximum pressure in the metatarsal region compared with maximum values in the heel region.

### 3.2. Planar Stride Strategy

Low control over the lower limb joints (hip, knee and ankle) reduces the active plantar zone and generates walking patterns in which high pressure is executed concurrently on the heel and forefoot. We defined two major parameters targeted to measure this strategy:

- Ratio between the forefoot pressure at the point of maximum heel pressure and the maximum forefoot pressure; and
- Anterior/posterior pressure pattern length.

Parameter A captures how much of the forefoot pressure is executed concurrently with heel pressure at the moment when heel pressure is more acute. Planar stride strategies will move forefoot pressure closer to heel pressure active intervals. Parameter B is based on the two-dimensional representation of the pressure patterns along the foot during the stance phase. The total anterior/posterior length will provide and overall estimation of the maximum flexion/extension moments in the joins. A reduced mobility in the lower limb joins will be translated into a shorter anterior/posterior pressure pattern as detected by the insole pressure sensors. 

### 3.3. Low Heel Pressure Strategy

Low coordination may generate walking patterns in which the initial foot ground contact point is not in the heel region. In many cases, the maximum pressure on the heel is very limited and the active region of the foot is limited to the forefoot and toes. Again, this strategy is due to a lack of dorsiflexion and reduced control at hip, knee and ankle. This could be either increased tone or reduced tone.

In order to evaluate this strategy, the following parameters have been defined:

- Ratio between the maximum forefoot pressure and the maximum heel pressure; and
- Anterior/posterior pressure length and location.

Parameter A provides a measure of relative pressure on the heel region in comparison with the forefoot. Parameter B captures the effective zone of plantar pressure used by the participant.

### 3.4. Gait Assymetries

Although major gait asymmetries tend to improve after rehabilitation, many stroke survivors walk using asymmetric strategies. Many parameters such as the length of strides, stance times or swing times have been used in literature. In this paper, we use some of the parameters defined for characterizing the previous strategies as the basis to calculate gait asymmetries. 

### 3.5. Gait Variability over Time

Some of the more severely affected users are not able to keep constant gait parameters over time. In this paper, we use some of the parameters defined for characterizing the previous strategies as the basis to calculate gait variability over time.

## 4. Results

This section presents the results obtained from the automatic data gathering and processing from the pressure insoles in order to automatically detect the walking strategies described in the previous section. Their detection is then fed into the self-rehabilitation system for gait re-learning. The final sub-section is dedicated to assess how well the RMI index could be determined based on the computed features. 

### 4.1. Heel Walking Strategy

This walking strategy consisted of long periods of high heel pressure vs. low maximum pressure exerted over the forefoot region.

**Figure 2:**
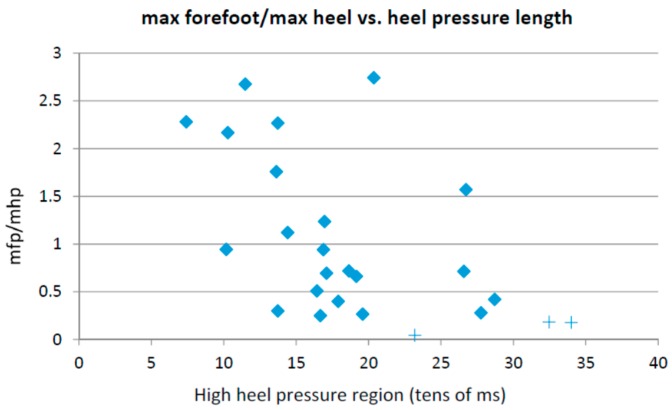
Maximum forefoot pressure vs. heel length.

Figure 2 shows a bi-dimensional plot of the period of time (in tens of milliseconds) in which the heel pressure is higher than 80% of its maximum value (in the *x* axis) vs. the quotient between the maximum forefoot pressure and the maximum heel pressure. Each point in the plot corresponds to the average step of each user at each foot. The “+” sign denotes the samples showing this particular walking strategy. 

**Figure 3:**
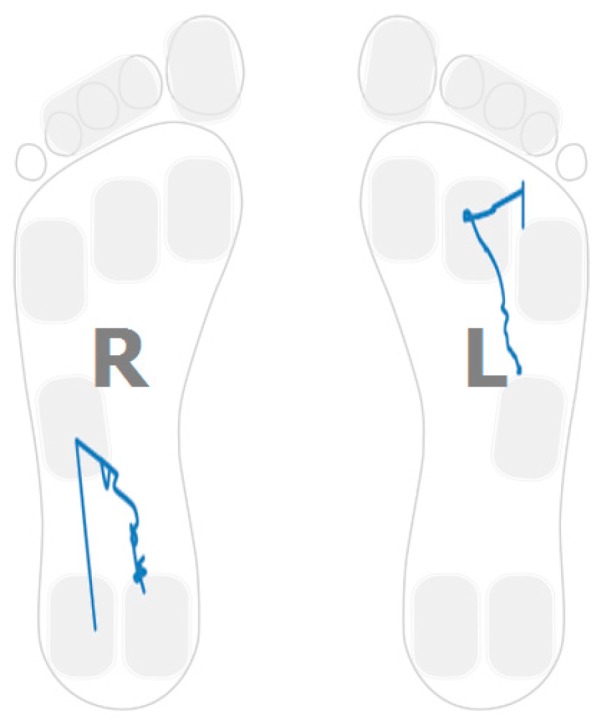
Post-stroke survivor. R: right; L: left.

**Figure 4:**
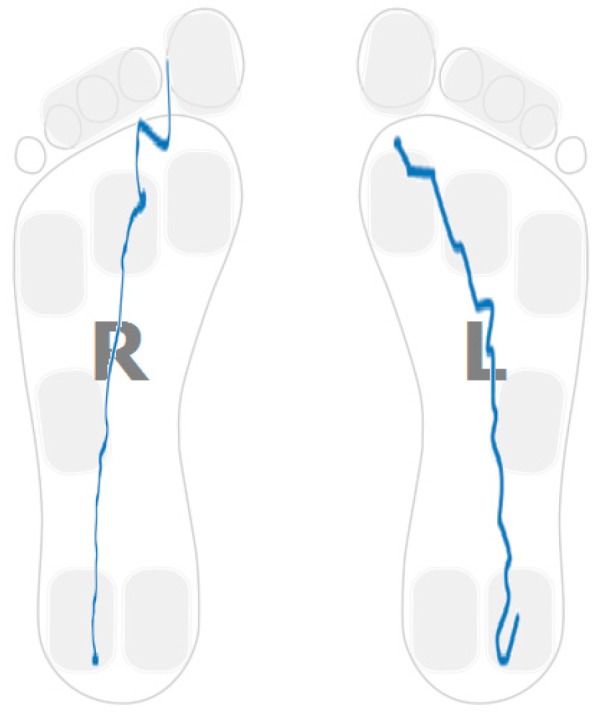
Healthy control.

The plantar pressure evolution plot for one of the participants showing this strategy in one foot is captured in Figure 3. In particular, the pressure pattern on the right foot is restricted to the heel area. The pattern shows a foot rotation over the heel region. Figure 4 captures a sample pattern for a healthy control user. The pressure plots for both feet go from heel to toe and distribute the person’s weight over the entire foot.

Using common classification techniques, patterns can be automatically classified as showing this walking strategy. Table 3 captures the results of applying the J48 classification algorithm to the data using a 10 fold cross validation technique to validate results (90% of the samples are used for training and 10% for validation, repeating the process 10 times until all samples have been in a validation set). All samples are assigned to the right class. 

### 4.2. Planar Stride Strategy

This walking strategy captured the cases in which high pressure is exerted on the forefoot region while the pressure on the heel is maximum. The results of applying EM clustering to the sensor data are captured in Table 4 (the mean and standard deviations for both clusters are shown). Two distinct clusters are found describing two different populations (*p* value for the *t*-test is 0.001196).

**Figure 5:**
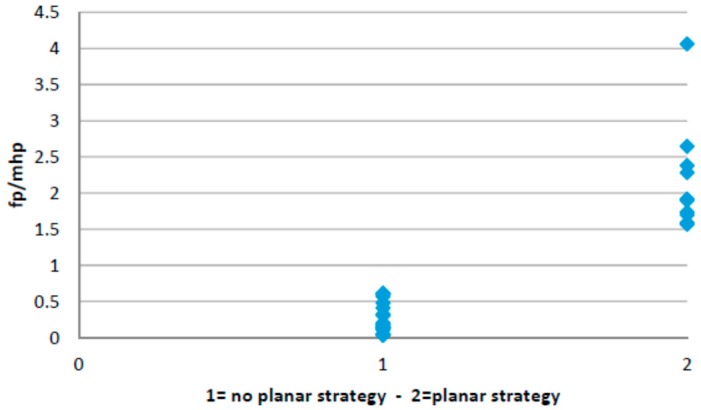
Sample distribution on both clusters.

**Figure 6:**
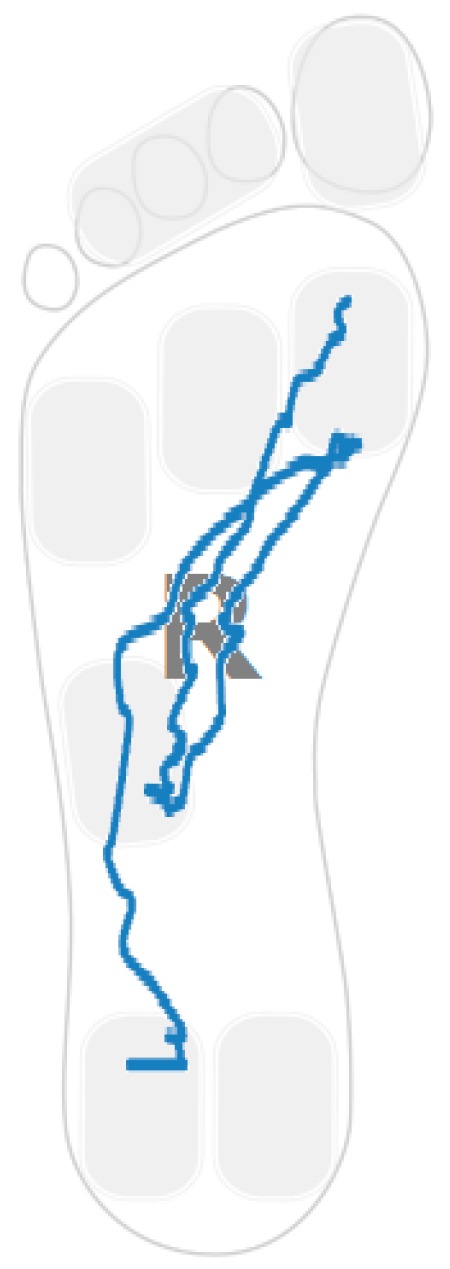
Pressure plot example.

Figure 5 shows the sample distribution using the ratio between forefoot pressure and heel pressure at maximum heel pressure point for data classified in each cluster. Figure 6 captures the distribution of the centre of pressure for one of the samples in the cluster showing this walking behaviour. A forward/backwards pattern is shown over the central part of the foot. Pressure patterns of a healthy control tend to capture a monotonic behaviour in the anterior-posterior axis (Figure 4). 

### 4.3. Low Heel Pressure Strategy

**Figure 7:**
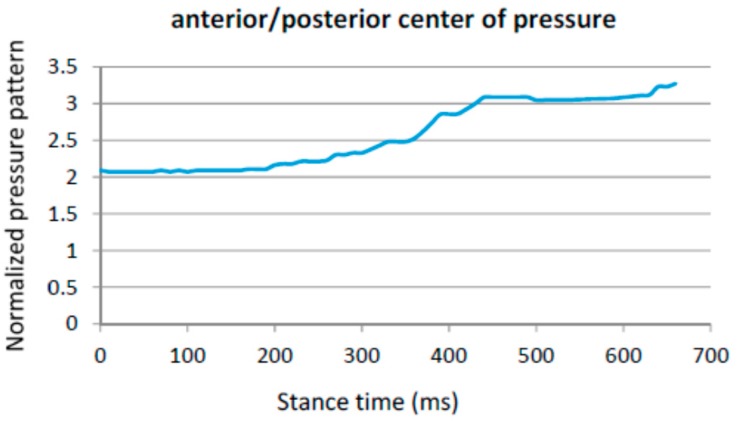
Anterior posterior centre of pressure over time (in milliseconds).

**Figure 8:**
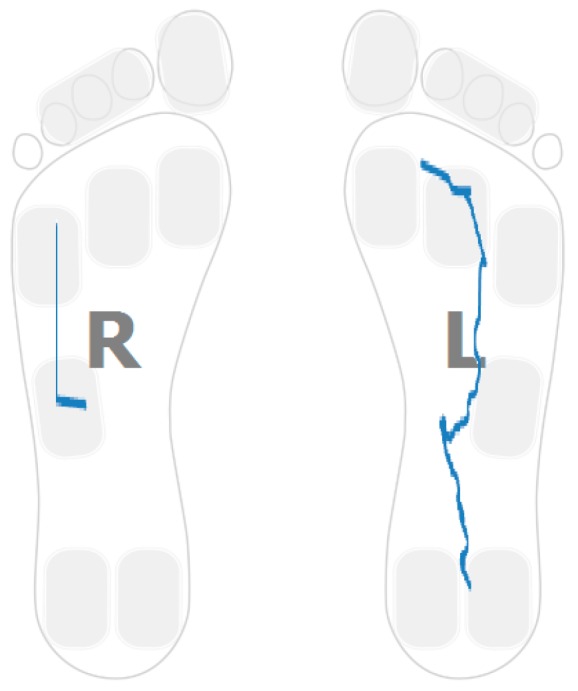
Post-stroke survivor pressure patterns.

**Figure 9:**
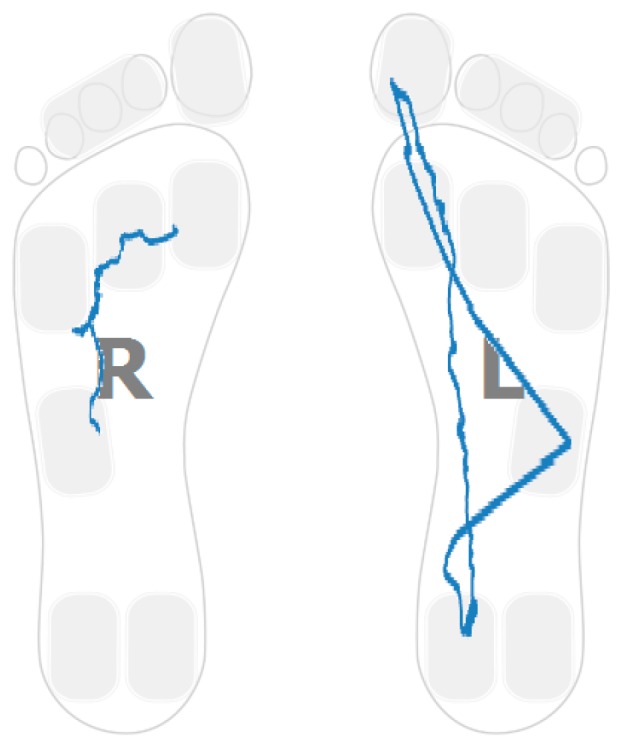
Post-stroke survivor pressure patterns.

The landing point in a healthy control is located in the heel region (Figure 4). For stroke-survivors, the landing part of the foot is not always in the heel region. Moreover, there are cases in which the user barely uses the heel region while walking. Figure 7 captures the anterior/posterior pressure graph for one of the participants using this walking strategy. The values have been normalized so that the heel region represents a value of “1” and the toes region represents a value of “4”. Figure 7 captures a pressure pattern that starts in the central part of the foot and covers up to the forefoot. The same pattern is mapped to the dimensional plantar plot in Figure 8 (in particular for the right foot). Figure 9 captures another example for another participant (this walking strategy can be seen for the right foot again). 

### 4.4. Gait Assymetries

**Figure 10:**
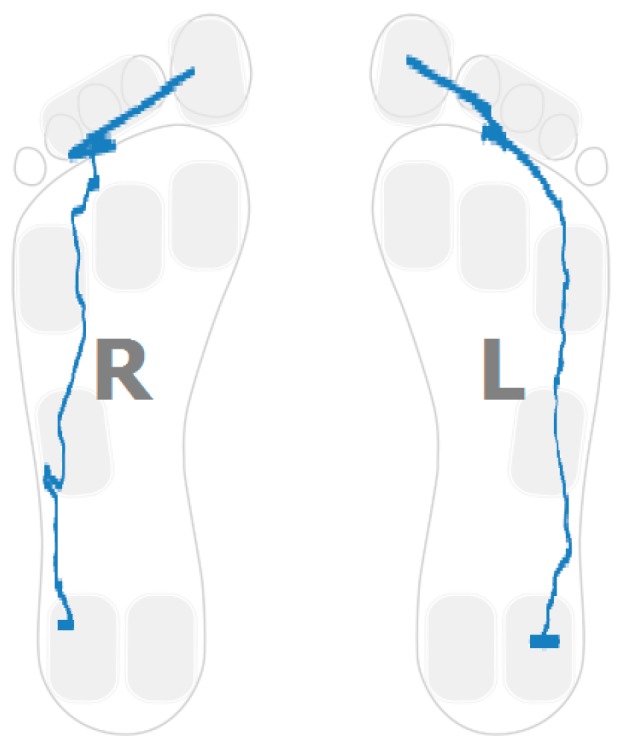
Pressure patterns for a stroke-survivor with RMI of 14.

Hemiplegia and hemiparesis after stroke translate into asymmetric gait patterns. Figure 3, Figure 6, Figure 8 and Figure 9 show pressure patterns with important asymmetries in both feet. After rehabilitation, some stroke survivors regain more symmetric gait patterns. Figure 10 captures the pressure patterns for both feet for a stroke survivor with a Rivermead Mobility Index of 14. 

**Figure 11:**
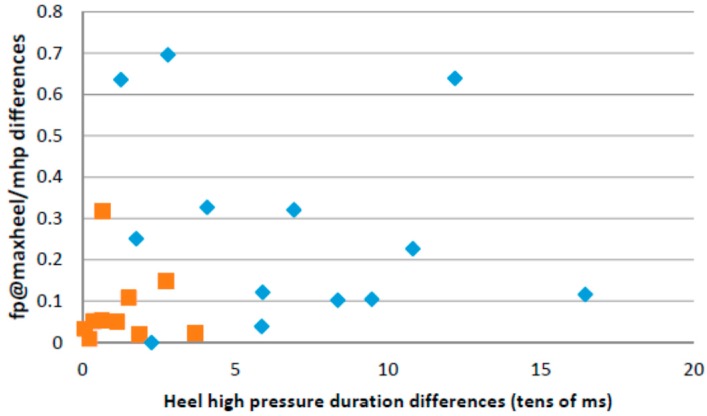
Heel pressure time difference vs. forefoot@maxheel/maxforefoot pressure differences between feet for both controls and stroke survivors.

Figure 11 captures two of the parameters used in this paper in a bi-dimensional plot for both stroke survivors and healthy controls. The *x*-axis captures the mean values for the heel pressure time differences between both feet, taking into account all the steps measured in the performed test for each person. The heel pressure time is the period of time in which the pressure on the heel region is higher than the 80% of its maximum value. The differences are in tens of milliseconds. The *y*-axis captures the mean values of the differences for both feet of the relative pressure calculated as the quotient between the forefoot pressure at the moment of maximum heel pressure and the maximum forefoot pressure. The healthy control group concentrates close to the origin while the samples corresponding to stroke survivors tend to at a further distance from the origin. 

### 4.5. Gait Variability over Time

**Figure 12:**
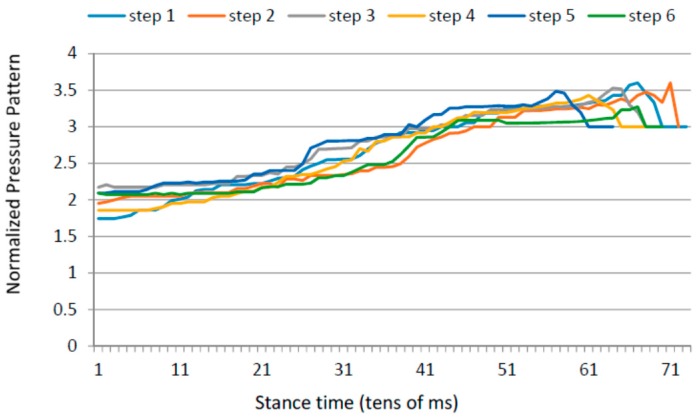
Anterior/posterior centre of pressure in the first six steps for a stroke survivor.

**Figure 13:**
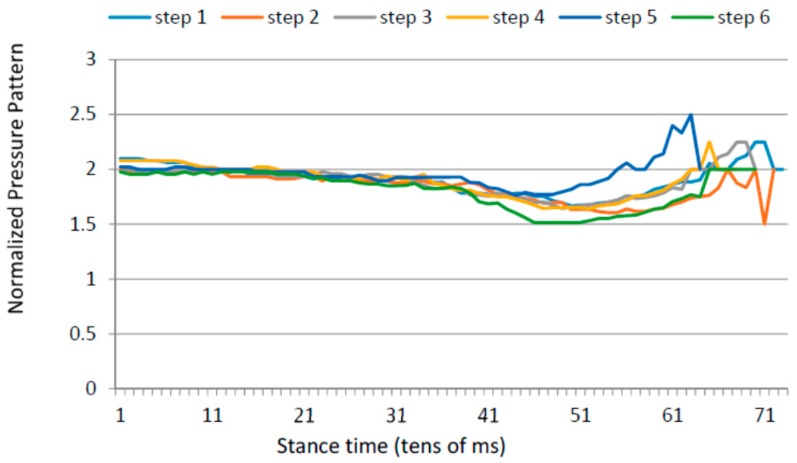
Lateral centre of pressure in the first six steps for a stroke survivor.

Apart from the inter-foot mean variation values, stroke survivors tend to show higher over time variability for each measured parameter (in both inter and intra-foot parameter variability over time). Figure 12 and Figure 13 show the anterior/posterior and lateral centres of pressure for the first six steps of a stroke survivor. The *x*-axis is the sample number counted from the floor landing instant (counted in tens of millisecond) and the *y*-axis is a linear scale from 1 to 4 for the anterior/posterior foot length (1 is assigned to the heel region and 4 represents the toes) and from 1 to 3 for the lateral foot length (1 being the inner foot and 3 the outer foot). Both variations in pressure values as well as in stride duration are captured. 

**Figure 14:**
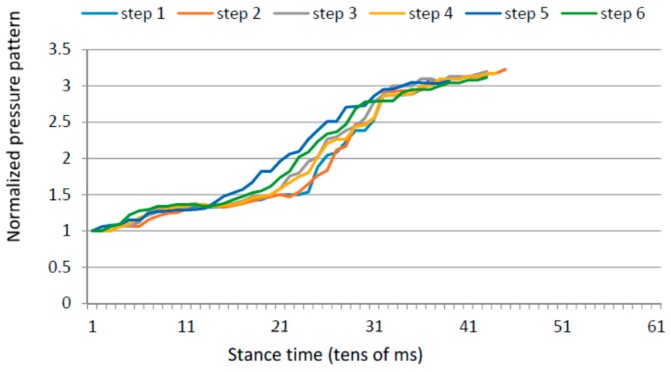
Anterior/posterior centre of pressure in the first six steps for a healthy control.

**Figure 15:**
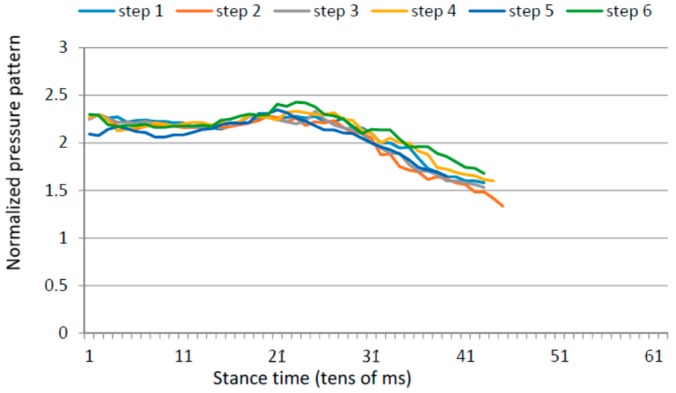
Lateral centre of pressure in the first six steps for a healthy control.

The same results for a healthy participant are presented in Figure 14 and Figure 15. The pressure values and durations show a more uniform pattern over time. 

To assess if differences in inter-stride variations for both populations (stroke survivors and healthy controls) are possibly caused due to random effects (null hypothesis) the *t*-test was used for two of the features computed in this study: the duration of heel pressure interval and the ratio between maximum pressures at the forefoot vs. the heel (Table 5). The standard deviation is computed for each parameter, each participant and each foot. In both cases, the *p*-value shows statistical significance under the 5% threshold so that the null hypothesis can be rejected. 

### 4.6. Correlations with the Rivermead Mobility Index

In order to support the development of an automatic assessment method based on the use of insole pressure sensors in the rehabilitation of stroke survivors, this section presents the correlation results between a human-assessed mobility index commonly used in rehabilitation (the Rivermead Mobility Index RMI) and some of the automatically computed features (based on the lateral and anterior posterior pressure patterns).

**Figure 16:**
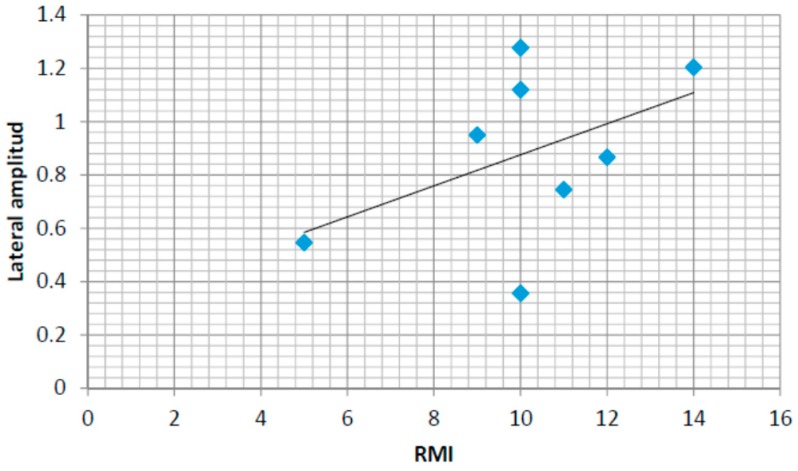
Rivermead Mobility Index (RMI) and lateral amplitude in the normalized pressure pattern.

Figure 16 shows the values for the mean lateral pressure variation amplitude for eight of the stroke survivors for which the RMI index have been also assessed. The pressure variation amplitude is calculated as the difference between the maximum and the minimum values for each step and the mean value is calculated considering all the steps for each person (for both feet). The lateral amplitude of the insoles has been normalized to values between 1 and 3. The maximum amplitude for lateral pressure variability is therefore normalized to a maximum value of 2. The correlation coefficient is r = 0.47. The coefficient of determination is r^2^ = 0.22. Although positive, only a moderate relationship could therefore be expected between the humanly assessed RMI index and the lateral pressure patterns.

**Figure 17:**
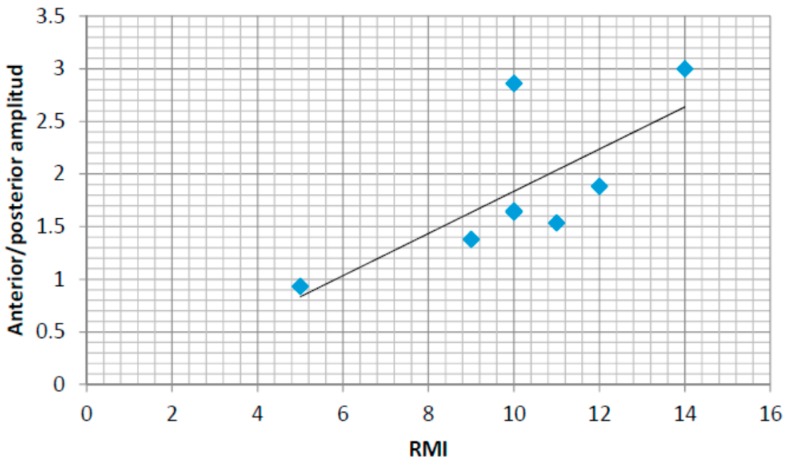
RMI and anterior/posterior amplitude in the normalized pressure pattern.

A similar analysis for anterior–posterior pressure patterns has also been performed. Figure 17 captures the same results for the anterior–posterior pressure values (normalized in this case for values from 1 to 4, 1 being the anterior/posterior location of the heel and 4 the anterior/posterior locations of the toes). The values for the linear regression are also shown in both figures showing a positive correlation between both automatically computed values from the pressure sensors and the human-assess RMI values. The normalized amplitudes in anterior/posterior and central/lateral pressure variations could be the basis for an autonomous assessment of the RMI index tool. In this case, the correlation coefficient is r = 0.73. The coefficient of determination is r^2^ = 0.53. Therefore, the RMI index can be better assessed automatically by using the anterior-posterior pressure patterns.

**Figure 18:**
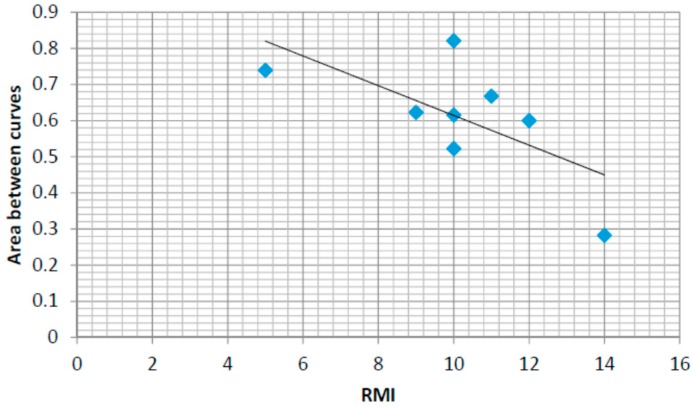
RMI and anterior/posterior vs. linear area between curves.

Another way we propose to automatically measure the rehabilitation progress of each individual is by comparing the pressure patterns with theoretical ones. Theoretical patterns can be obtained by modelling the values obtained from healthy control users. For example, if we define a model that characterizes the anterior posterior centre of pressure following a linear-like pattern for healthy people from heel to toe, we train this model with data from healthy people and we calculate the difference between the theoretical curve in the model and the real one computed for the insole pressure sensors we get the following expression:

(1) $$\bar{ABC}=\frac{1}{N}\overset{N}{\underset{i=1}{\sum }}abs\left(C_{i}-P_{i}\right)$$

where $\bar{ABC}$ represents the Area Between Curves divided by the duration of the foot ground-contact interval, N is the number of samples in the average ground contact zone, $C_{i}$ are the values for the real calculated pressure pattern and $P_{i}$ represents the values for the theoretical pattern. Figure 18 shows the results calculated for the same eight participants whose RMI values where measured. There is a negative correlation between the progress in rehabilitation and the difference between curves showing that the rehabilitation process brings patients towards the theoretical pattern. The error values for the healthy controls group after the training of the model are around 0.2. In this case, the correlation coefficient is r = 0.51. The coefficient of determination is r^2^ = 0.26.

## 5. Discussions and Conclusions

Following the development of the PSMrS with integrated data capture via an intelligent sensored insole, we aimed to explore, in a pilot study, whether the automatic computation of insole-data based features could detect some of the common characteristics and walking strategies adopted by a small cohort of stroke survivors. The automatically computed parameters identified in this study will contribute to the on-going development of a technology enhanced personalized self-management rehabilitation system for stroke survivors. This automation in the perception and assessment is the basis for personalized feedback and is a keystone in achieving four key sources of self-efficacy (mastery experiences, modelling, interpreting physiological signs and feedback and persuasion). 

The heel walking strategy generated ground contact pressure patterns that were assessed by calculating the heel contact length and the ratio between forefoot and heel maximum pressure. The 2D plots of the centre of pressure distribution during the stance phase clearly showed the part of the foot used and the limitations in mobility for each person. 

The planar stride strategy was evaluated. Simultaneous high pressure on the heel and the forefoot regions as well as 2D pressure plots were able to provide valuable information in order to assess the degree of rehabilitation. 

For stroke survivors using a ground first contact point outside the heel, a low heel pressure strategy was defined and evaluated. The anterior/posterior pressure plots were shown to be a good tool for evaluating this strategy. 

Gait asymmetries and gait variability over time were assed demonstrating how to classify participants in both groups, which were the normal values and variations in the healthy control group and the major differences stroke group. The *p*-value has been used to assess if differences are happening by chance (null hypothesis) or not (under a 5% significance threshold value).

Finally, correlations between the Rivermead Mobility Index and the anterior-posterior normalized pressure amplitude and the area between pressure curves comparing a linear model, fit for the control group, and the data from each stroke survivor have been studied. Using a linear regression model, a positive correlation has been found between the RMI index and the anterior-posterior amplitude and a negative correlation between the area under curve and the RMI index. The central-lateral amplitude shows a weaker correlation with the RMI index.

This study has confirmed that stroke survivors utilize a number of strategies in order to walk post stroke, and that the insole used to gather data within the PSMrS can accurately record these characteristics within these strategies. We believe these data can now be translated into feedback screen on a mobile device, providing some of the key sources of self-efficacy that can contribute to motor relearning and recovery. Following this, a randomized controlled trial will be undertaken to investigate the effectiveness of the novel PSMrS for stroke survivors. 

## Figures and Tables

**Table 1:** Stroke survivor.

Unique ID	Gender	Age (years)	Affected Side	Insole Size	Weight (kg)
11	F	64	Right	L	105
12	M	61	Right	L	85
13	F	66	Right	M	75
15	M	50	Left	XL	90
16	F	79	Left	S	64.8
21	F	72	Right	M	73
22	M	64	Left	XL	90.8
23	F	75	Right	L	114.3
24	M	75	Left	L	80
25	F	68	Both sides	M	95.3
26	F	69	Left	M	66
27	M	84	Right	XL	95.3
29	M	39	Both sides	L	84.1
210	M	64	Right	XL	87.3

**Table 2:** Healthy participants.

Unique ID	Gender	Age (years)	Insole Size	Weight (kg)
NKP1	F	45	M	63.5
NKP4	M	44	L	69.9
NKP5	F	46	S	64.8
NKP6	F	55	M	64.1
NKP7	F	54	M	75
NKP9	M	45	XL	80
NKPA	F	52	S	64.2
NKPB	F	50	M	72
NKPC	M	46	M	70
NKPD	F	51	S	54

**Table 3:** J48 classification results.

Classified as	Heel Walking	No Heel Walking
Heel walking	3	0
No heel walking	0	21

**Table 4:** EM clustering results.

Cluster	Mean	Standard Deviation
1	2.0906	0.9036
2	0.279	0.2033

**Table 5:** *T*-test results.

Feature	*p*-Value
heel duration standard deviation	0.0161
Forefoot vs. heel maximum pressure standard deviation	0.0138
